# Atypical Presentation of Coxiella burnetii Endocarditis: Diagnostic Considerations and the Importance of Keeping a Broad Differential

**DOI:** 10.7759/cureus.63659

**Published:** 2024-07-02

**Authors:** Michael Fragner, Sudarshan S Srivats, Kevin Pink, Hassan Abuhashish

**Affiliations:** 1 Internal Medicine, New York-Presbyterian Brooklyn Methodist, Brooklyn, USA; 2 Internal Medicine, Catholic Medical Center, Manchester, USA; 3 Cardiology, New York-Presbyterian Brooklyn Methodist, Brooklyn, USA

**Keywords:** aortic valve infective endocarditis, prosthetic valve infective endocarditis, q-fever, culture negative infective endocarditis, coxiella burnetti

## Abstract

Coxiella burnetii is a gram-negative bacterium associated with serious complications such as infective endocarditis. Early diagnosis and treatment can be difficult due to its nonspecific presentation and risk factors that include contact with farm animals or their byproducts. Here, we present an atypical presentation of infective endocarditis caused by Coxiella burnetii, where the patient had no risk factors, negative Duke criteria, and negative preliminary workup.

## Introduction

Infective endocarditis (IE) is characterized by bacterial colonization on the endocardial surfaces of cardiac valves or, in some cases, intracardiac devices. Common organisms in IE include Staphylococcus and Streptococcus; however, Coxiella burnetii (C. burnetii) can be the culprit on rare occasions with an incidence of 1 in 1,000,000 [1]. The bacterium responsible for "Q fever," C. burnetii, is a gram-negative organism primarily transmitted to humans through contact with infected animals such as cattle, sheep, goats, or even dogs in some instances [2,3]. Symptoms are nonspecific and include fever, fatigue, night sweats, weight loss, and heart murmurs [4].

Although early recognition and treatment are crucial to avoid potential complications, the diagnosis of C. burnetii IE can be challenging due to its nonspecific symptoms and slow-growing nature, leading to treatment delays and poor prognosis [5]. Clinicians should consider C. burnetii IE in patients presenting with nonspecific symptoms when they have had previous exposure to farm animals or their byproducts. It is vital to recognize the importance of considering Q fever in patients with culture-negative endocarditis and prosthetic heart valves. This report was previously presented as a meeting abstract at the 2024 ACC Annual Scientific Session in April 2024 and is greatly expanded on, demonstrating a rare presentation of IE caused by C. burnetii, which was identified early and successfully treated.

## Case presentation

A 58-year-old Dominican male, with a history of hypertension, hyperlipidemia, non-insulin-dependent diabetes, heart failure with preserved ejection fraction due to ischemic cardiomyopathy, implantable cardioverter defibrillator (ICD), and triple vessel disease requiring coronary artery bypass graft (CABG) using a saphenous vein graft to the left anterior descending artery, presented for intermittent chills and rigors over the past several months. He also has a history of aortic stenosis requiring a bioprosthetic aortic valve replacement (AVR). This was complicated by prosthetic valve endocarditis (PVE) requiring sternotomy and mechanical AVR with an ascending aorta conduit.

Three weeks prior to this admission, he presented with similar symptoms with additional pleuritic chest pain. Evaluation at that time was unremarkable, and he was discharged home without antibiotics or new medications. Of note, his last visit to the Dominican Republic was more than 10 years ago where he worked as a police officer. Since being in Brooklyn, he has worked in construction. He denied any exposure to animals besides a dog for a brief period over 10 years ago. On admission, he was hemodynamically stable and afebrile. A physical exam demonstrated a well-appearing, obese male with evidence of a systolic murmur at the second right intercostal space. There was no evidence of lower extremity edema, respiratory distress, or volume overload.

**Figure 1:**
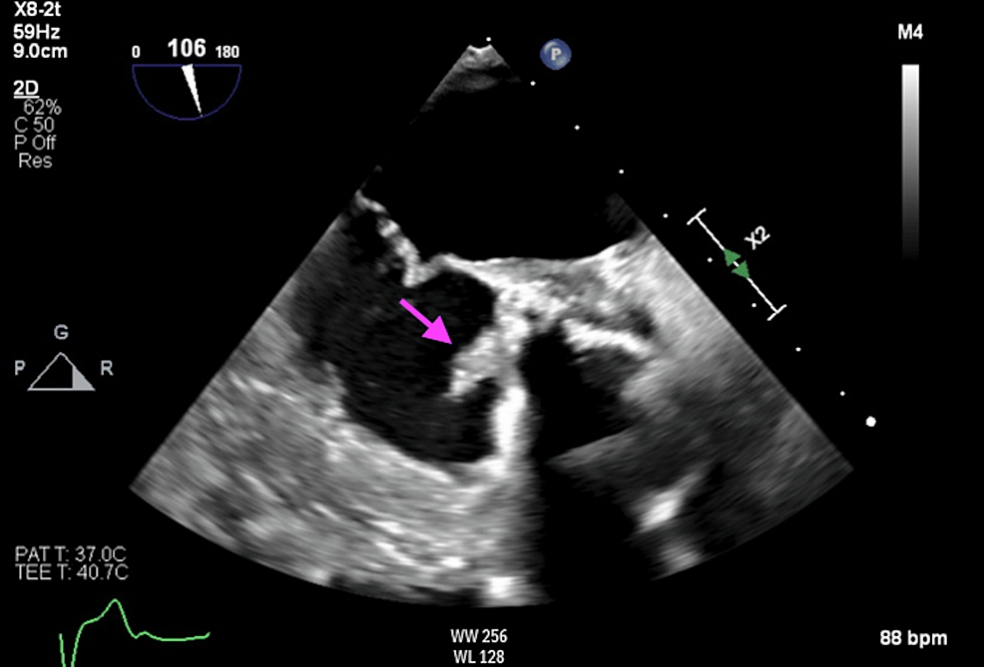
Transesophageal echocardiogram mid-esophageal view Arrow indicating aortic root thickening and irregular vegetation on the ventricular aspect of the posterior aortic valve ring extending to the aortomitral continuity, consistent with a para-valvular abscess.

Laboratory values on admission were notable for a white blood cell count of 10,220/microliter, erythrocyte sedimentation rate of 113 mm/hr, C-reactive protein of 45.2 mg/L, pro-brain natriuretic peptide (pro-BNP) of 423 pg/mL, and two negative high sensitivity troponin values. The electrocardiogram on admission showed sinus rhythm with a first-degree AV block and left-axis deviation. Given the concern for the recurrence of infective endocarditis, the patient was admitted to the cardiology floor and started on intravenous vancomycin and ceftriaxone empirically for broad-spectrum antibiotic coverage. A transesophageal echocardiogram showed an ejection fraction of 60% and a large, irregular, mobile vegetation on the ventricular aspect of the posterior aortic valve ring extending to the aortomitral continuity. These findings were consistent with paravalvular abscess (Figure 1).

Due to the patient’s subacute presentation, there was concern for a possible underlying immunocompromised state, such as human immunodeficiency virus, which was ruled out. His history and risk factor of a mechanical valve raised suspicion for infective endocarditis. 

**Figure 2:**
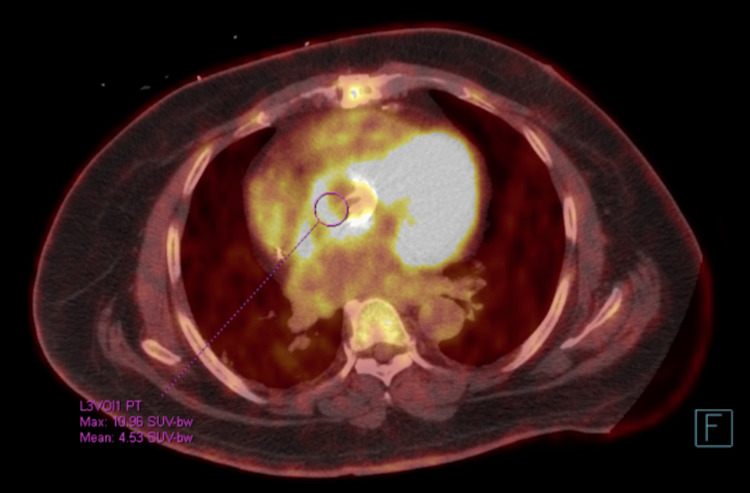
Fluorodeoxyglucose positron emission tomography/computed tomography (FDG PET/CT) Irregular thickening and intense FDG avidity of the mechanical aortic valve and aortic root, suspicious for infectious/inflammatory etiology

Cardiac, chest, and abdominal CT angiography were performed, which revealed diffuse thickening of the aortic root. Serial blood cultures collected prior to antibiotics were performed and remained negative throughout the hospital stay; however, serum serology returned positive with elevated immunoglobulin G (IgG) titers for Coxiella burnetii (1:131072). These findings were concerning for Coxiella burnetii IE, and his antibiotics were switched to doxycycline and he was started on hydroxychloroquine. During the patient’s hospital stay, telemetry noted frequent bouts of nonsustained ventricular tachycardia and prolongation of the PR interval on daily EKGs (300-340 milliseconds) concerning for involvement of the conduction system. Follow-up fluorodeoxyglucose positron emission tomography/computed tomography (FDG PET/CT) imaging showed irregular thickening and intense radioactive tracer avidity of the mechanical aortic valve and root (Figure 2).

The patient was evaluated for extraction of his ICD and re-operative AVR conduit. Cardiac catheterization was completed showing a patent graft to the left anterior descending artery and normal left and right heart pressures. The patient subsequently underwent ICD lead extraction after completion of three weeks of intravenous antibiotics. Soon after ICD lead extraction, the patient had a re-operative AVR conduit and root replacement without complication.

## Discussion

Coxiella burnetii (Q fever) is a rare cause of IE. The etiology includes contact of skin or mucous membranes with infected animal tissue or fluid, inhalation of infected aerosolized particles, or consumption of unpasteurized dairy products; the latter being the most common means of transmission [6]. Acute Q fever typically presents as fever, headache, and cough, allowing the diagnosis to be easily missed, as it masquerades as a typical viral illness. If not treated, acute Q fever can progress to chronic Q fever months to years after initial infection [4]. In our case, the peculiarity of the patient is that he never had notable risk factors that would make one suspect Q fever. However, given no obvious source of infection with negative blood cultures, Coxiella burnetii was tested. An uncommon manifestation of chronic Q fever is IE, which is seen in immunosuppressed patients or those with prosthetic heart valves or vascular abnormalities [7]. Those with prosthetic valves are at higher risk compared to those with native valvular anomalies. More often the mitral and aortic valves are affected. Coxiella burnetii endocarditis should be suspected if there are clinical and echocardiographic findings suggesting endocarditis, and if blood cultures are negative within 72-96 hours [8].

Diagnosis is made via serological testing using indirect immunofluorescence assay (IFA) that detects antibodies in serum, both IgM and IgG [8]. There is an antigenic phase I and antigenic phase II to which antibody responses are developed. During an acute infection with Q fever, Coxiella burnetii phase II antigen predominates, while in chronic infection, the phase I antigen is higher and more often IgG. IgM antibodies will rise with IgG antibodies; however, IgM will remain elevated for longer periods of time and have an increased rate of cross-reactivity with other species such as Bartonella and Legionella. Thus, IgM titers have a lower specificity in diagnosis than IgG, but both IgM and IgG titers should be tested to obtain serologic diagnosis. IgG titers are used to detect chronic Q fever. Using Duke criteria for IE, an IgG antibody titer >1:800 is deemed a major criterion. If this is seen, the performance of a transthoracic echocardiogram (TTE) is indicated for further evaluation [9,10]. Additionally, polymerase chain reaction (PCR) can be used for diagnosis but is more sensitive in the first two weeks of illness (acute phase) and within 24-48 hours of antibiotic administration. Thus, a negative PCR test does not rule out the diagnosis and serological testing should be performed [8].

Imaging has emerged as an innovative tool in the diagnosis of Coxiella burnetii infection, especially for infective endocarditis. Specifically, FDG-PET/CT has become increasingly useful in localizing foci of infection due to the ability of inflammatory cells to uptake a significant amount of FDG. This is especially important in Q fever due to its broad predilection for affecting multiple organs at once [11]. In Eldin et al.'s retrospective study of 167 patients who were diagnosed with C. burnetii infection and had undergone FDG-PET/CT, 21 patients were suggested to have endocarditis. Interestingly, in this subgroup prior to the study, 13 patients were determined to possibly have IE, 5 were not suspected to have IE at all, and only 3 had definite IE. Six cases were on native valves, 14 were on prosthetic valves, and 1 was on a pacemaker [11]. By retrospectively analyzing 25 patients with confirmed IE (18 prosthetic valves, 7 native valves), Ricciardi et al. showed that although TTE had a higher sensitivity for the detection of IE as compared to FDG-PET/CT overall (80% vs 55%), FDG-PET/CT was better at detecting PVE compared to TTE. In this study, 16/18 PVE cases were detected using FDG-PET/CT. By examining the cases that underwent transesophageal echocardiography, sensitivity for detecting PVE was greatest utilizing FDG-PET/CT (85%) as compared to echocardiography (69%) and Duke criteria (77%) [12]. Given the propensity for C. burnetii IE to present clandestinely, and often without typical features of endocarditis such as vegetations, FDG-PET/CT has emerged as a resourceful option to enhance diagnosis. This is especially true for PVE.

The mainstay of treatment of chronic Q fever with endocarditis is with doxycycline, 100 mg every 12 hours, and hydroxychloroquine, 200 mg every 8 hours, for a minimum of 18 months or up to 24 months in patients with prosthetic valves [13]. Duration of treatment is based on serologic response via IgG titers and with clinical improvement. Frequent monitoring of serology is required during treatment due to the possibility of a relapse of the disease if treatment is stopped too soon [14]. In a case series described by Sanchez-Recalde et al., 20 patients with Coxiella burnetii IE were studied, of which 14 patients had prosthetic heart valves, 6 had native heart valves, and 15 of the 20 ultimately required valve replacement [15]. Additionally, it is imperative to note the mortality rate is higher in patients with Q fever IE as compared to patients with Q fever but without complications. In a study by van Roeden et al. examining a cohort of patients with chronic Q fever, the mortality rate was demonstrated to be an extraordinary 38% with a median follow-up of 3.7 years, further highlighting the severity of Q fever IE [16].

## Conclusions

IE is associated with a high mortality rate, especially in patients with PVE complicated by paravalvular invasion. In suspected endocarditis, it is imperative to keep a broad differential diagnosis. Judicious and timely diagnosis of IE using the modified Duke criteria allows for prompt identification of the suspected microorganism and initiation of appropriate antibiotics. It is vital to understand the potential complications associated with Coxiella burnetii IE, such as paravalvular abscess formation and involvement of the conduction system, and the need for timely surgical intervention in severe cases. Clinicians should also understand the high mortality rate in Q fever IE compared to Q fever without complications and the importance of regular follow-up. Coxiella burnetii should be strongly considered as part of the differential diagnosis when suspecting IE, especially in patients with risk factors presenting with an insidious course.
